# Genetic prediction of male pattern baldness based on large independent datasets

**DOI:** 10.1038/s41431-022-01201-y

**Published:** 2022-11-07

**Authors:** Yan Chen, Pirro Hysi, Carlo Maj, Stefanie Heilmann-Heimbach, Timothy D. Spector, Fan Liu, Manfred Kayser

**Affiliations:** 1 Department of Genetic Identification, Erasmus MC, University Medical Center Rotterdam, Rotterdam, The Netherlands; 2 Key Laboratory of Genomic and Precision Medicine, Beijing Institute of Genomics, Chinese Academy of Sciences, Beijing, China; 3 University of Chinese Academy of Sciences, Beijing, China; 4 Department of Twin Research and Genetic Epidemiology, King’s College London, London, United Kingdom; 5 Institute for Genomic Statistics and Bioinformatics (IGSB), University of Bonn, School of Medicine & University Hospital Bonn, Bonn, Germany; 6 Institute of Human Genetics, University of Bonn, School of Medicine & University Hospital Bonn, Bonn, Germany

**Keywords:** Genetics, Genetic markers

## Abstract

Genetic prediction of male pattern baldness (MPB) is important in science and society. Previous genetic MPB prediction models were limited by sparse marker coverage, small sample size, and/or data dependency in the different analytical steps. Here, we present novel models for genetic prediction of MPB based on a large set of markers and large independent subsample sets drawn among 187,435 European subjects. We selected 117 SNP predictors within 85 distinct loci from a list of 270 previously MPB-associated SNPs in 55,573 males of the UK Biobank Study (UKBB). Based on these 117 SNPs with and without age as additional predictor, we trained, by use of different methods, prediction models in a non-overlapping subset of 104,694 UKBB males and tested them in a non-overlapping subset of 26,177 UKBB males. Estimates of prediction accuracy were similar between methods with AUC ranges of 0.725–0.728 for severe, 0.631–0.635 for moderate, 0.598–0.602 for slight, and 0.708–0.711 for no hair loss with age, and slightly lower without, while prediction of any versus no hair loss gave 0.690–0.711 with age and slightly lower without. External validation in an early-onset enriched MPB dataset from the Bonn Study (*N* = 991) showed improved prediction accuracy without considering age such as AUC of 0.830 for no vs. any hair loss. Because of the large number of markers and the large independent datasets used for the different analytical steps, the newly presented genetic prediction models are the most reliable ones currently available for MPB or any other human appearance trait.

## Introduction

Male pattern baldness (MPB), or androgenetic alopecia, is a highly heritable, age independent appearance trait [1, 2]. MPB has socio-psychological consequences on a large proportion of the world’s male population. MPB is of interest to various fields of scientific research and applications, such as in medicine, anthropology, evolutionary biology, and forensics. Availability of accurate and reliable genetic prediction of MPB would allow addressing several concerns. Genetic MPB prediction could enable early treatment of MPB before it becomes externally manifest, which may result in better therapeutic outcomes alleviating socio-psychological problems. MPB prediction from old or modern DNA would help describing the appearance of historical persons from skeletal remains or DNA found at crime scenes, as previously done for pigmentation traits [3, 4]. MPB prediction from ancient DNA would allow reconstructing what ancestors of modern humans looked like and help understanding when and where MPB first arose in time and space, as previously shown for pigmentation traits [5, 6]. Additionally, as MPB was discussed as marker of several later-onset health disorders (e.g., metabolic and cardiovascular diseases), genetic MPB prediction may contribute to better management of age-dependent human diseases.

A key requirement for the development of accurate genetic prediction models for genetically complex traits like MPB is the availability of sufficiently large number of DNA predictors. To date, four sufficiently powered GWASs for MPB have identified hundreds of MPB-associated SNPs, available for MPB prediction modelling. Heilmann-Heimbach et al. [7] reported a GWAS meta-analysis of 10,846 early-onset MPB cases and 11,672 controls from eight independent cohorts and identified 63 distinct genetic loci. Hagenaars et al. [8] identified 287 genetic loci in 52,874 UK Biobank (UKBB) male participants. Pirastu et al. [9] described 71 genetic loci in 43,590 UKBB male participants, follow by an independent replication study in 31,112 men. Recently, Yap et al. [2] identified 624 genetic loci from what currently is the largest GWAS on MPB, involving 205,327 UKBB males.

A key requirement for developing reliable genetic prediction models for MPB and any other trait are sufficiently large and independent datasets needed for the different analytical steps. The genetic prediction models by Hagenaars et al. [8], which with 331 SNPs currently include the largest number of DNA predictors, discriminated between subjects with no hair loss from those with severe hair loss with an AUC 0.78 (sensitivity = 0.74, specificity = 0.69, PPV = 0.59, NPV = 0.82). Although these models were tested in independent dataset of 12,874 UKBB samples, GWAS-based marker discovery and prediction model training were performed in the very same dataset of 40,000 UKBB samples, and no external model validation was reported. It remains unclear, if and to what degree the non-independence of datasets for marker discovery and model training induces bias and how they would perform in the external validation in different datasets.

Aiming to overcome limitations of previously reported models, we developed new genetic prediction models for MPB based on a large number of DNA markers and four large independent datasets for the four analytical steps i.e., i) 55,573 UKBB samples for prediction marker ascertainment, ii) 104,694 UKBB samples for prediction model training, iii) 26,177 UKBB samples for prediction model testing, and iv) 991 samples from the early-onset MPB enriched Bonn Study for external model validation.

## Materials and methods

### UK Biobank Study dataset

The UK Biobank Study (http://www.ukbiobank.ac.uk) is a large, population-based genetic epidemiology cohort of volunteers living in the UK. In response to UK Biobank question 2395 [8], male participants chose one answer that most closely matched their head hair coverage from four given patterns adapted from the Hamilton-Norwood scale [10, 11] (Supplementary Fig. 2), i.e., no, slight, moderate, and severe hair loss (Supplementary Fig. 2). Details on microarray genotyping and imputation have been described previously [12]. After genomic and phenotypic quality controls, to avert issues of population admixture and stratification, the current study included a total of 186,444 UKBB males of European descent, divided into three independent subsets for: 1) prediction marker ascertainment (*N* = 55,573), 2) model training (*N* = 104,694), and 3) model testing (*N* = 26,177) (Supplementary Fig. 1). The 80–20% data split into model training and model testing datasets resulted in the same proportions of each of the four baldness phenotype categories observed over the combined dataset (Table 1). Our prediction marker ascertainment dataset used for feature selection likely included all the 52,874 UKBB men previously used in the GWAS by Hagenaars et al. [8]. We initially considered a total of 270 out of the original Hagenaars et al. [8] 287 MPB-associated SNPs at 85 genetic loci which were carried forward in our feature selection analysis (Supplementary Table 1). The rest were removed due to our quality control procedures. This loss is due to an up todate genotypic and imputated SNPs.

**Table 1:** Characteristics of study samples.

		UK Biobank Study			Bonn Study
		Feature selection	Model training	Model testing	External model validation
Age	Number of males	55,573	104,694	26,177	991
	Mean/Sd (all)	57.23/8.02	57.00/8.12	56.93/8.15	46.45/16.38
	Mean/Sd (No hair loss)	56.19/8.18	55.82/8.30	55.75/8.35	66.45/6.81
	Mean/Sd (Slight hair loss)	55.68/8.32	55.49/8.32	55.46/8.41	57.80/7.18
	Mean/Sd (Moderate hair loss)	58.82/7.47	58.73/7.53	58.61/7.55	43.75/14.25
	Mean/Sd (Severe hair loss)	58.65/7.48	58.44/7.61	58.41/7.61	34.60/6.26
Hair Loss	No hair loss	31.95%	31.98%	31.98%	31.69%
	Slight hair loss	22.94%	23.18%	23.18%	1.61%
	Moderate hair loss	26.78%	26.58%	26.58%	16.65%
	Severe hair loss	18.33%	18.26%	18.26%	50.05%
	Any hair loss	68.05%	68.02%	68.02%	68.31%

### Bonn Study dataset

The Bonn Study is a case-control study designed to investigate the genetic basis of early-onset MPB. This study includes 991 males of European descent from Germany i.e., 582 early-onset MPB cases representing the 20% most severely affected in the respective age-groups (<30 years; <40 years), 268 “unaffected” controls ≥60 years with no signs of baldness representing the 10% least affected in their age-group, and 141 samples from the population-based Heinz Nixdorf Recall cohort. Hair loss status was ascertained by expert dermatologists according to the Hamilton/Norwood (HN) classification [11, 13]. Baldness status was aligned to the same categories as done in the UKBB Study (Supplementary Fig. 2). Details on microarray genotyping and SNP imputation have been described previously [7]. Of the 117 SNP predictors we identified via feature selection in UKBB, 107 were available in the Bonn dataset. The 10 SNPs missing in Bonn were neither present in the genotyping array dataset nor could they be properly imputed. These data were used as external model validation dataset to additionally test the MPB prediction models trained and tested in UKBB data.

### Multinomial and binomial logistic regression

The multinomial logistic regression (MLR) and binomial logistic regression (BLR) modelling was implemented via *multinom* function in ‘*nnet’* package of R (www.r-project.org). The modelling specifications were as following. For MLR, consider baldness pattern, y, to be four categories: i) no hair loss, ii) slight hair loss, iii) moderate hair loss and iv) severe hair loss, which are determined by the genotype, *x*, of *k* SNPs, where *x* represents the number of minor alleles. If *π*_1_,*π*_2_,*π*_3_, and *π*_4_ denote the probability of the four MPB categories, respectively, the MLR can be written as

$${{{{{{{\mathrm{logit}}}}}}}}\left( {\Pr \left( {{{{{{{{\mathrm{y}}}}}}}} = {{{{{{{\mathrm{no}}}}}}}}\;{{{{{{{\mathrm{hair}}}}}}}}\;{{{{{{{\mathrm{loss|}}}}}}}}x_1 \ldots x_k} \right)} \right) = \ln \left( {\frac{{\pi _1}}{{\pi _4}}} \right) = \alpha _1 + \mathop {\sum }\limits_{i = 1}^k \beta (\pi _1)_ix_i$$

$${{{{{{{\mathrm{logit}}}}}}}}\left( {\Pr \left( {{{{{{{{\mathrm{y}}}}}}}} = {{{{{{{\mathrm{slight}}}}}}}}\;{{{{{{{\mathrm{hair}}}}}}}}\;{{{{{{{\mathrm{loss|}}}}}}}}x_1 \ldots x_k} \right)} \right) = \ln \left( {\frac{{\pi _2}}{{\pi _4}}} \right) = \alpha _2 + \mathop {\sum }\limits_{i = 1}^k \beta (\pi _2)_ix_i$$

$${{{{{{{\mathrm{logit}}}}}}}}\left( {\Pr \left( {{{{{{{{\mathrm{y}}}}}}}} = {{{{{{{\mathrm{moderate}}}}}}}}\;{{{{{{{\mathrm{hair}}}}}}}}\;{{{{{{{\mathrm{loss|}}}}}}}}x_1 \ldots x_k} \right)} \right) = \ln \left( {\frac{{\pi _3}}{{\pi _4}}} \right) = \alpha _3 + \mathop {\sum }\limits_{i = 1}^k \beta (\pi _3)_ix_i$$

where α and β can be derived in the modelling set. Baldness pattern of each individual in the testing set can be probabilistically predicted based on the genotypes and the derived α and β,

$$\pi _1 = \frac{{{{{{{{{\mathrm{exp}}}}}}}}(\alpha _1 + \mathop {\sum }\nolimits_{i = 1}^k \beta (\pi _1)_ix_i)}}{{1 + \exp \left( {\alpha _1 + \mathop {\sum }\nolimits_{i = 1}^k \beta \left( {\pi _1} \right)_ix_i} \right) + \exp \left( {\alpha _2 + \mathop {\sum }\nolimits_{i = 1}^k \beta \left( {\pi _2} \right)_ix_i} \right) + \exp \left( {\alpha _3 + \mathop {\sum }\nolimits_{i = 1}^k \beta \left( {\pi _3} \right)_ix_i} \right)}}$$

$$\pi _2 = \frac{{\exp \left( {\alpha _2 + \mathop {\sum }\nolimits_{i = 1}^k \beta \left( {\pi _2} \right)_ix_i} \right)}}{{1 + \exp \left( {\alpha _1 + \mathop {\sum }\nolimits_{i = 1}^k \beta \left( {\pi _1} \right)_ix_i} \right) + \exp \left( {\alpha _2 + \mathop {\sum }\nolimits_{i = 1}^k \beta \left( {\pi _2} \right)_ix_i} \right) + \exp \left( {\alpha _3 + \mathop {\sum }\nolimits_{i = 1}^k \beta \left( {\pi _3} \right)_ix_i} \right)}}$$

$$\pi _3 = \frac{{\exp \left( {\alpha _3 + \mathop {\sum }\nolimits_{i = 1}^k \beta \left( {\pi _3} \right)_ix_i} \right)}}{{1 + \exp \left( {\alpha _1 + \mathop {\sum }\nolimits_{i = 1}^k \beta \left( {\pi _1} \right)_ix_i} \right) + \exp \left( {\alpha _2 + \mathop {\sum }\nolimits_{i = 1}^k \beta \left( {\pi _2} \right)_ix_i} \right) + \exp \left( {\alpha _3 + \mathop {\sum }\nolimits_{i = 1}^k \beta \left( {\pi _3} \right)_ix_i} \right)}}$$

$$\pi _4 = 1 - \pi _1 - \pi _2 - \pi _3$$

The baldness category with the max(*π*_1_, *π*_2_, *π*_3_, *π*_4_) was considered as the predicted baldness pattern.

For binomial logistic regression (BLR), consider baldness pattern, y, to be two categories any hair loss and no hair loss, which are determined by the genotype, *x*, of *k* SNPs, where *x* represents the number of minor alleles. Let *p* denote the probability of any hair loss, and 1 − *p* is the probability of no hair loss. The BLR can be written as

$${{{{{{{\mathrm{logit}}}}}}}}\left( {\Pr \left( {y = {{{{{{{\mathrm{any}}}}}}}}\,{{{{{{{\mathrm{hair}}}}}}}}\,{{{{{{{\mathrm{loss}}}}}}}}\left| {x_1 \ldots x_k} \right.} \right)} \right) = \ln \left( {\frac{p}{{1 - p}}} \right) = \alpha + \mathop {\sum}\limits_{i = 1}^k {\beta _ix_i}$$

where α and β can be derived in the modelling set. Baldness pattern of each individual in the testing set can be probabilistically predicted based on his or her genotypes and the derived α and β,

$$p = \frac{{\exp \left( {\alpha + \mathop {\sum}\nolimits_{i = 1}^k {\beta _ix_i} } \right)}}{{1 + \exp \left( {\alpha + \mathop {\sum}\nolimits_{i = 1}^k {\beta _ix_i} } \right)}}$$

The MPB category with the max(*p*, 1 − *p*) was considered as the predicted baldness pattern.

### Support vector machine and Artificial neural network

Next to logistic regression analyses, two types of machine learning (ML) algorithms were additionally applied for prediction modelling: support vector machine (SVM) and artificial neural networks (ANN). SVM was used via *svm* function in the e1071 package of R with the nonlinear radial basis function as the kernel and 2^-4^ as the cost of constraints violation set. A “one against one” strategy was applied for classification of both 4-category and 2-category baldness pattern, and posterior probability estimates for each baldness category were obtained via *predict.svm* function in the e1071 R package. A feed-forward ANN was implemented in Tensorflow [14] and Keras (https://github.com/fchollet/keras). The ANN model consisted of three layers with a hidden layer of ten neurons using the Rectified Linear Unit (ReLU) function in hidden layer and softmax activation function in output respectively. The output layer contained four neurons for 4-category baldness pattern and two neurons for binary baldness pattern, each represents yes-no for one baldness type. The training algorithm of the neural network was performed using Adam optimizer [15]. ANN model training and testing were conducted in Python version 3.6.1 using the package keras. As for the hyper parameter tuning, we used grid search and chosen hyper parameter with the best prediction performance for both ANN and SVM. For ANN model, we considered activation function of tanh, relu and sigmoid, the number of neurons in hidden layer ranging from 10 to 117 (including 10, 20, 30, 40, 50, 60 and 117), and patience of 25, 50 and 100. For SVM, we considered kernel of linear, polynomial, radial and sigmoid, cost of 2^−5^, 2^−4^, 2^−3^, 2^−2^, 2^−1^, 1, 2, 2^2^, 2^3^ and 2^4^. We got the best hyper parameters and built best model by using these best hyper parameters, that is, ANN model using activation function of relu, 10 neurons, and patience of 100, and best SVM model using kernel of radial and cost of 2^−4^.

For both SVM and ANN models, the input data included 118 features (age and genotypes at the 117 SNP marker sites) and output was the probability of being classified into each baldness pattern category. The same 80% training and 20% testing datasets as applied for regression analyses were also used for SVM and ANN analyses. The derived SVM and ANN models were subsequently used to predict baldness pattern categories in the model testing dataset, returning 4-element and 2-element probability vectors for 4-category and binary baldness pattern with values between 0 and 1, respectively. The baldness category with the maximal value was considered as the predicted category.

### Feature selection, prediction model training, and model testing

We conducted a Forward Stepwise Regression (FSR) analysis in the marker ascertainment dataset of UKBB by iteratively including the next largest contributor to the model according to Akaike Information Criterion (AIC), ${{{\rm{AIC}}}} \sim 2k + n{\rm ln} (\mathop{\sum}\limits^{n}_{i=1} (y_i-E(y_i))^2)$, where *k* is the number of markers, *n* is the sample size, and $E\left( {y_i} \right)$ is the fitted value. FSR was performed on 4-category MPB phenotypes using MLR. The marker set proposed by FSR with the minimal AIC value was considered as the theoretically optimal subset for prediction analysis. The rank of the SNP predictors was determined by their rank in FSR analysis or their association significance.

Prediction models were built in the prediction model training dataset of UKBB using MLR and BLR considering MPB as a 4-category as well as as binary trait by joining all samples into two categories of no hair loss and any hair loss. Additionally, SVM and ANN models also were built in the same datasets and using the same four and two categories, respectively. The developed prediction models were assessed for their accuracy in the model testing dataset of UKBB and additionally externally validated in the Bonn Study dataset.

Prediction accuracy was evaluated by estimating the following parameters including the Area Under the Receiver Operating Characteristic (ROC) Curves (AUC), sensitivity, specificity, positive prediction value (PPV), and negative prediction value (NPV). AUC is the integral of ROC curves which ranges from 0.5 representing random prediction to 1.0 representing perfect prediction. Sensitivities and specificities, PPVs and NPVs were calculated using confusion matrices considering the highest predicted probability as the predicted baldness pattern for 4-category prediction, while for binary prediction considering the predicted probability >0.5 as the predicted baldness pattern. Statistical analyses and result visualisation were conducted in R version 3.6.1 using nnet, e1071, pscl, and pROC packages.

## Results

### Phenotypic characteristics in UKBB and Bonn Study

Individual phenotype and genotype data of 187,435 men from two European countries were included i.e., 186,444 males from the UKBB (32% no, 23% slight, 27% moderate, and 18% severe hair loss; 57.05 ± 8.10 years of age; age range 39–73) and 991 males from the Bonn Study (2% slight, 16% moderate, and 50% severe hair loss; 46 ± 16 years of age; age range 18–70; Table 1, Supplementary Figs. 1 and 2). In the UKBB, age was significantly associated with increased MPB severity (Supplementary Fig. 3). In the Bonn Study, lower mean ages were observed across the four MPB categories, because a large proportion (58.7%) of the participants were early-onset MPB (Supplementary Fig. 3).

### Feature selection, prediction model training, and model testing in UKBB data

**Fig. 1:**
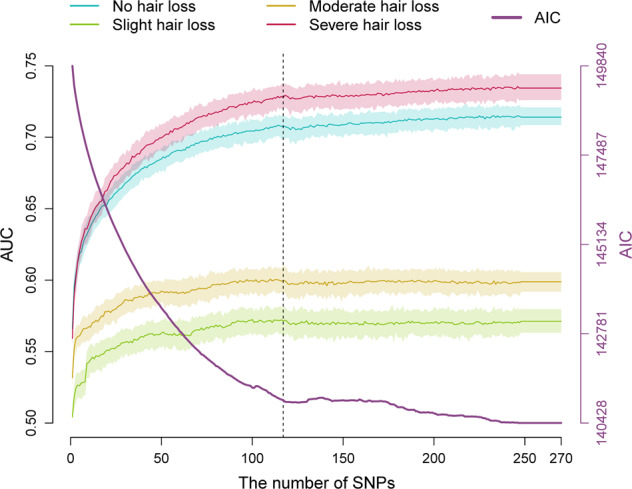
Relationship between the number of SNP predictors and the prediction accuracy expressed as AUC and AIC for 4-category MPB prediction in the marker ascertainment dataset of 55,573 UKBB males. This dataset includes the 52,874 UKBB men previously applied for discovering the MPB-association of the 270 SNPs [8] used here for feature selection. Based on this dataset, a MLR model was fit in a randomly selected 80% prediction model training subset and applied in the remaining 20% prediction model testing subset with 100 replicates. The estimated mean AUCs are depicted as coloured line and the 5–95% boundary as respective light colour shade for the four predicted MPB categories. The dashed line marks the cut-off at 117 SNPs that were used as best-fit prediction marker set in all subsequent prediction analyses.

Feature selection (or feature thinning) of 270 MPB-associated SNPs from 85 genetic loci previously discovered in the study of Hagenaars et al. [8] was carried out in 55,573 UKBB samples using FSR. Notably, this sample set likely includes all the 52,874 UKBB men previously used by Hagenaars et al. (Supplementary Table 1) for GWAS-based discovery of these 270 associated SNPs. This analysis identified 101 SNPs from 69 distinct loci, which in multinomial logistic combination achieved the maximal model fitting. To this FSR-selected prediction marker set, we additionally added the top-associated SNP from each of the remaining 16 genetic loci (Supplementary Table 1), resulting in a total set of 117 SNP predictors from all 85 distinct loci (Pseudo Nagelkerke-*R*^*2*^ = 0.22). The remaining 153 SNPs from these loci that were removed from our models by FSR did not independently contribute to the MBP prediction (Fig. 1), justifying their exclusion.

**Fig. 2:**
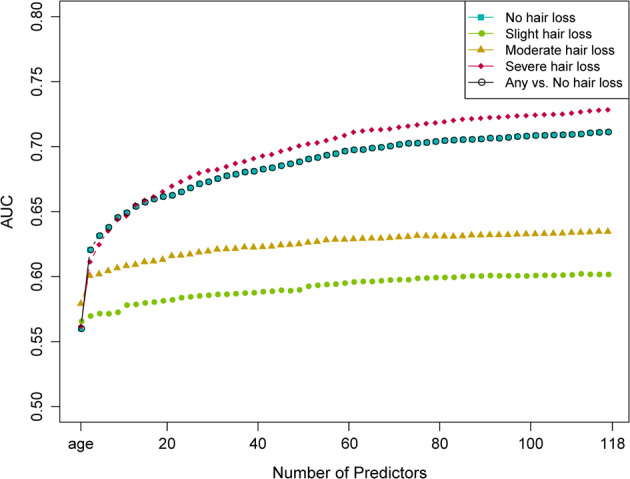
Individual contribution of the ascertained 117 SNP predictors and age on the prediction accuracy expressed as AUC for the 4-category MLR and the 2-category BLR MPB prediction models obtained from the model testing dataset of 26,177 UKBB men. These prediction models were built in the prediction model training dataset of 104,694 UKKBB men.

We then used these 117 SNP predictors with and without considering age as additional predictor to build 4-category (no, slight, moderate, and severe hair loss) MLR models as well as 2-category (no *versus* [vs.] any hair loss) BLR models in the model training subset of 104,694 UKBB males. Subsequently, these models were validated in the independent model testing subset of 26,177 UKBB males and prediction accuracy parameters were estimated (Supplementary Table 2). Accumulative AUC analysis in the model testing subset showed a fast to slow increase according to the ranking of the predictors (Fig. 2). Notably, AUC increased at faster rates for comparisons of no hair loss with increasingly severe hair loss categories. As expected for an age-dependent trait, age was the strongest single predictor, alone resulting in an AUC of 0.560 for any vs. no hair loss, followed by the 101 SNPs selected by the FSR analysis, then followed by the extra 16 SNPs. The most predictive SNP was rs12558842 on the X chromosome, which is located ~280 kbp upstream of the androgen receptor (AR) gene. Alongside age, rs12558842 provided an AUC of 0.608 for any vs. no hair loss. The MBP-association of rs12558842 was stronger than that of rs2497938 in the study of Hagenaars et al., and the later was the highest ranking MPB predictor in Liu et al. [16]. Rs2497938 is in perfect linkage equilibrium with rs2207081, which fully masks the predictive power of the polyglycine GGN triplet repeat of *AR* [17].

**Fig. 3:**
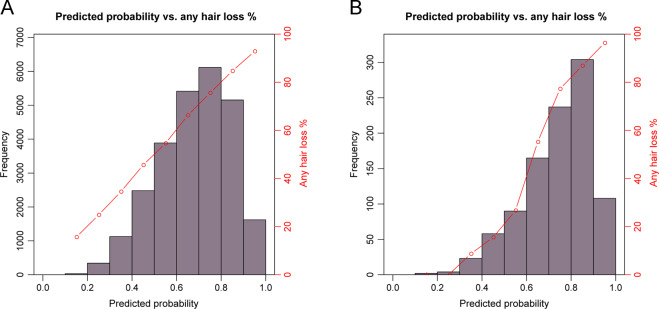
Histograms of predicted probability overlaid with percentage of MPB categories (including slight, moderate and severe hair loss) in each probability bin. **A** The probability was derived from MPB prediction model with 117 SNPs and age as predictors in the prediction model testing dataset of 26,177 UKBB men. These prediction results were practically informative for about 26.2% of the tested males (0.4% <0.2 and 25.8% >0.8). **B** The probability was derived from MPB prediction model with 107 SNPs as predictors without age in the external validation dataset of 991 men from the Bonn Study. These prediction results were practically informative for about 41.8% of the tested males (0.2% <0.2 and 41.6% >0.8). These 117-SNP (**A**) and the 107-SNP (**B**) MPB prediction models were built in the prediction model training dataset of 104,694 UKBB men.

The 4-category MLR models without considering age predicted with considerably higher accuracies for severe (AUC = 0.718) and no hair loss (0.702) than for moderate (0.607) and slight (0.574) hair loss (see Table 2 for additional prediction accuracy metrics). Inclusion of age in the MLR modelling improved the estimates of accuracy to AUC of 0.728 for severe hair loss, 0.711 for no hair loss, 0.635 for moderate hair loss, and 0.602 for slight hair loss (Table 2). For the 2-category BLR model of predicting any vs. no hair loss, we obtained an AUC of 0.702 without age and a slightly higher AUC of 0.711 with age in the model (Table 2). This model provided informative prediction for about 26.2% of the 26,177 males tested, that is, individuals with predicted probabilities of any hair loss <0.2 or >0.8 (Fig. 3A).

**Table 2:** Accuracy estimates of genetic prediction of categorical MPB obtained from the prediction model testing dataset of 26,177 UKBB men using different methods based on 117 SNP predictors with and without age as additional predictor^a^.

			No hair loss	Slight hair loss	Moderate hair loss	Severe hair loss	Any vs. no hair loss
Logistic regression	Without age	AUC	0.702	0.574	0.607	0.718	0.702
		SENS	0.702	0.071	0.379	0.318	0.917
		SPEC	0.590	0.949	0.736	0.891	0.255
		PPV	0.446	0.298	0.342	0.395	0.724
		NPV	0.808	0.772	0.766	0.854	0.591
	With age	AUC	0.711	0.602	0.635	0.728	0.711
		SENS	0.679	0.122	0.408	0.309	0.911
		SPEC	0.616	0.925	0.741	0.901	0.278
		PPV	0.454	0.331	0.363	0.412	0.728
		NPV	0.803	0.778	0.776	0.854	0.595
Artificial neural network	Without age	AUC	0.696	0.571	0.602	0.713	0.687
		SENS	0.678	0.116	0.329	0.356	0.899
		SPEC	0.608	0.914	0.775	0.870	0.277
		PPV	0.449	0.290	0.346	0.380	0.726
		NPV	0.801	0.774	0.761	0.858	0.563
	With age	AUC	0.709	0.598	0.634	0.728	0.706
		SENS	0.646	0.164	0.424	0.292	0.915
		SPEC	0.651	0.899	0.726	0.911	0.267
		PPV	0.465	0.328	0.359	0.423	0.727
		NPV	0.797	0.781	0.777	0.852	0.598
Support vector machine	Without age	AUC	0.699	0.568	0.603	0.715	0.676
		SENS	0.699	0.086	0.352	0.329	0.930
		SPEC	0.586	0.942	0.756	0.881	0.206
		PPV	0.442	0.307	0.343	0.382	0.713
		NPV	0.805	0.773	0.763	0.855	0.578
	With age	AUC	0.708	0.598	0.631	0.725	0.690
		SENS	0.675	0.134	0.394	0.310	0.924
		SPEC	0.619	0.917	0.750	0.896	0.230
		PPV	0.454	0.327	0.364	0.400	0.719
		NPV	0.802	0.778	0.774	0.853	0.589

^a^These models were built in the prediction model training dataset of 104,694 UKBB men.

We further investigated the performance of the MLR models over six age groups of the model testing subset, i.e. in men 39–45, 46–50, 51–55, 56–60, 61–65, and 66–72 years of age. Overall, the performance of the predictive models with and without age was similar across these six age groups and the four different MPB categories (Supplementary Fig. 4). Slight differences were seen for some MPB categories and some age groups, such as better performance of the model with age for no hair loss in 39–45 years old and for moderate hair loss in 39–45 and 46–50 years old men.

The SVM and ANN machine-learning models provided similar prediction accuracy estimates compared to the M/BLR models (Table 2). As with M/BLR models, we also saw for SVM and ANN models that when including age, the prediction accuracy estimates were higher than without age in the models. Notably, seeing similar prediction estimates of accuracy from M/BLR and SVM/ANN methods in such large datasets may imply a lack of epistasis or nonlinear associations. It is unclear if this finding is a reflection of the true genetic architecture of MPB, or may be the consequence of the additive, univariate model used in the SNP discovery GWAS, whose results were carried forward into the ascertainment of the SNP predictors used in the models.

### External model validation in Bonn Study data

External validation was conducted in 991 males from the Bonn Study (58.7% early-onset cases, 27% unaffected controls, and 14.2% were population-based). Due to differences in genotyping platforms and quality control analyses, 10 of the 117 SNPs (Supplementary Table 3) were unavailable in the Bonn Study. Therefore, we first trained the models based on the 107 SNPs available in Bonn by using the UKBB training subset and validated them in UKBB testing subset. This demonstrated only marginally lower performance of the 107-SNP models compared to the full 117-SNP models in UKBB (AUC loss 0.002–0.006, Table 3).

**Table 3:** Accuracy estimates of genetic prediction of categorical MPB obtained from the prediction model testing dataset of 26,177 UKBB men and the external validation dataset of 991 Bonn men with and without age as predictor using Multinomial and Binominal Logistic Regression based on 107 of the 117 SNP predictors available in Bonn^a^.

			No hair loss	Slight hair loss	Moderate hair loss	Severe hair loss	Any vs. no hair loss
UKBB testing dataset	Without Age	AUC	0.696	0.573	0.602	0.712	0.696
		SENS	0.697	0.072	0.373	0.313	0.919
		SPEC	0.579	0.951	0.738	0.893	0.244
		PPV	0.438	0.309	0.340	0.394	0.721
		NPV	0.803	0.773	0.765	0.853	0.586
	With Age	AUC	0.705	0.600	0.631	0.722	0.705
		SENS	0.678	0.120	0.409	0.301	0.915
		SPEC	0.614	0.926	0.737	0.904	0.265
		PPV	0.452	0.329	0.360	0.412	0.726
		NPV	0.802	0.777	0.775	0.853	0.594
Bonn external validation dataset	Without Age	AUC	0.830	0.599	0.591	0.775	0.830
		SENS	0.694	0	0.430	0.444	0.984
		SPEC	0.818	0.963	0.695	0.857	0.245
		PPV	0.639	0	0.220	0.756	0.738
		NPV	0.852	0.983	0.859	0.606	0.875
	With Age	AUC	0.559	0.694	0.505	0.539	0.550
		SENS	0.500	0.067	0.193	0.144	0.928
		SPEC	0.704	0.648	0.827	0.911	0.147
		PPV	0.436	0.003	0.180	0.623	0.704
		NPV	0.755	0.978	0.838	0.509	0.484

^a^These models were built in the prediction model training dataset of 104,694 UKBB men.

In the Bonn Study, the 107-SNP models that did not include age as predictor, achieved considerably higher prediction accuracy estimates compared to UKBB i.e., 0.830 in Bonn vs. 0.696 in UKBB for no hair loss, 0.775 vs. 0.712 for severe hair loss, and 0.830 vs. 0.696 for any vs. no hair loss. For the slight hair loss phenotype, the AUC in Bonn was slightly higher than in UKBB with 0.599 vs. 0.573, while for moderate hair loss it was slightly lower with 0.591 vs. 0.602 (Table 3). For any vs. no hair loss prediction, the BLR model without considering age in Bonn provides accurate prediction for about 41.8% of males, that is, individuals with predicted probabilities of any hair loss <0.2 or >0.8 (0.2% <0.2 and 41.6% >0.8, Fig. 3B).

For the 107-SNP models that included age as predictor, we achieved considerably less accurate prediction performances in the Bonn Study compared to UKBB (Table 3) i.e., AUC of 0.559 in Bonn vs. 0.705 in UKBB for no hair loss, 0.539 vs. 0.722 for severe hair loss, 0.550 vs. 0.705 for any vs. no hair loss, and 0.505 vs. 0.631 for moderate hair loss. For slight hair loss, however, it was higher in Bonn than in UKBB with 0.694 vs. 0.600. For the BLR model predicting any vs. no hair loss, the sensitivity was similarly high with (0.928) and without (0.984) age in Bonn and higher in Bonn than in UKBB (0.915 and 0.919) (Table 3). The relative drop in performance of age-inclusive models in the Bonn Study is likely a consequence of age confounding which arises from its case-control design. The deliberate focus in the recruitment of early MBP cases and relatively old super-controls results in a very different age distribution and a negative correlation between MBP and age in this cohort, as opposed to the expected positive correlations expected in the wider population as seen in UKBB.

## Discussion

Reliable genetic predictions for MPB can contribute to different disciplines of scientific research and science applications, including in medicine, evolutionary biology, anthropology, human history, and forensics. Previously reported genetic prediction models for MPB were limited by sparsity of genetic marker coverage, low-powered sample size, and non-independence of data used in the different analytical steps of prediction modelling. Here, we have sought to overcome these previous limitations by developing and validating new genetic MPB prediction models based on a large number of DNA predictors and large and fully independent datasets in the different analytical steps. These new models achieve more accurate and more reliable genetic MPB prediction than previous models.

Previously, Marcińska et al. [18] applied 20 SNPs from only 10 MPB-associated loci and achieved an AUC of 0.66 to discriminate between male subjects with vs. without significant baldness, in a model that did not include age as a predictor. The prediction accuracy we achieved here with our 117-SNP model without age for any vs. no hair loss was considerably higher (AUC = 0.702) than that reported by Marcińska et al. [18]. This improvement is likely due to the effect of the largely increased number of SNP predictors that we included in our models by taking advantage of recently published GWAS-based marker knowledge.

For another previous genetic MPB prediction model based on 11 SNPs from only 7 genetic loci plus age, Liu et al. [16] reported an AUC of 0.711 for no hair loss vs. any hair loss, which surprisingly was the same as what we achieved with our 117-SNP model with age (Table 2). Aiming to understand this apparent similarity of AUC while using more than 100 additional DNA preditors, we build a MPB prediction model in the UKBB model training subset based on 10 available of the 11 SNPs used by Liu et al. [16]. Considering age as additional predictor, this 10-SNP model achieved an AUC of 0.611 in the UKBB model testing subset (Supplementary Table 4), considerably lower than the AUC = 0.711 reported by Liu et al. for all 11 SNPs [16]. This 0.1 difference in AUC cannot be explained by the one missing SNP duplicated? in this comparative analysis. Rather, this may be explained by the larger effect of age on MPB prediction in the Rotterdam Study [16] (a contribution of 0.171 to the AUC), an ageing cohort with mean age of 68 years, considerably older than the UKBB (mean age 57) where age contributes only 0.06 to the AUC. Alternatively, higher prediction accuracies may result from the better investigator-conducted phenotyping in the Rotterdam Study compared with the self-reported UKBB phenotyping.

The recently reported genetic prediction model for MPB by Hagenaars et al. based on 331 SNPs [8], including the 270 SNPs we used in our feature selection analysis (the identity of only these 270 SNP predictors out of the 331 was publicly available at the time of writing) was built in 40,000 UKBB participants' samples that were also used for GWAS-based marker discovery. This model was internally validated in 12,874 additional UKBB samples by Hagenaars et al. and achieved AUCs of 0.78 for no vs. severe hair loss, 0.68 for no vs. moderate hair loss, and 0.61 for no vs. slight hair loss without considering age, and 0.79, 0.70 and 0.61, respectively, for models that additionally included age [8]. A direct comparison of these AUC estimates with the AUCs from our 4-category approach is difficult. In our analysis, we considered all individuals from all four categories to estimate AUC per each category, while Hagenaars et al. left out data that did not fit the predicted categories. Although we believe that using all data is the better way, we attempted a direct comparison by estimating the prediction accuracy of a model based on our 117 SNP predictors using the same analytical approach as Hagenaars et al. [8]. In our UKBB model testing subset, this analysis revealed AUC values of 0.81 for no vs. severe hair loss, 0.73 for no vs. moderate hair loss, and 0.63 for no vs. slight hair loss when age is included as a predictor, which were higher than those previously reported by Hagenaars et al. This different prediction performance may be explained by the larger model training dataset we used here, which generally increases the predictive performance of statistical models. These results, however, provide no evidence that the Hagenaars et al. approach of performing marker discovery and model training in the very same dataset led to over-inflated prediction accuracy.

Our study also demonstrates that it still is a long way towards highly accurate genetic prediction of MPB such as already available for eye colour with AUCs of 0.94 [3, 4]. As next further step, all of the 624 MPB-associated loci identified in the entire UKBB data resource of >200,000 male subjects [2] shall be used for prediction modelling. This, however, can only be done once sufficiently large independent datasets for model training, testing, and external validation become available in the future. It is possible though, as implied by the results of our feature selection analysis, that inclusion of more SNPs may not necessarily lead to the envisioned significant improvement of MPB prediction accuracy, unless perhaps a large number is added. This is because GWASs with larger sample size are known to discover associated SNPs with smaller allelic effect sizes, which are expected to have smaller predictive value compared to SNPs previously identified in smaller-sized GWASs included in the current models. Although the new prediction models that we introduce here can reliably predict a good proportion of MPB risk in subjects of European ancestry and thus have good potential for practical use, future models may benefit from additional predicting biomarkers, for which also other types of biomarkers, such as epigenomic and metabolomic biomarkers, should be investigated.

In summary, we introduce new genetic prediction models for MPB that overcome limitations of previous models by including a large number of genetic predictors and utilizing large and independent datasets for the different analytical steps. Therefore our new models can be considered the most reliable genetic prediction models available to date for MPB and any other human appearance trait.

## Supplementary information

Supplementary Information

## Data Availability

The UK Biobank data are available from the UK Biobank (https://www.ukbiobank.ac.uk/).
