# Supplementary material for: Genetic prediction of male pattern baldness based on large independent datasets
Source: Eur J Hum Genet. 2022 Nov 7;31(3):321–8. doi: 10.1038/s41431-022-01201-y (PMC9995341; doi:10.1038/s41431-022-01201-y)

Supplementary Information

Genetic prediction of male pattern baldness based on large independent datasets

Supplementary Table 1. Association results of 270 SNPs* considering MPB as a continuous variable in 55,573 UKBB males used here as feature selection dataset.

SNP	CHR	BP	EA	P	Locus
rs142020459	1	10572083	G	2.50E-08	1
rs17035390	1	10653622	C	3.71E-08	1
rs2242288	1	10684098	A	6.95E-09	1
rs59304342	1	10753094	A	2.40E-08	1
rs7542354	1	11040385	A	3.65E-61	1
rs143916866	1	24967537	T	2.05E-07	2
rs1005734	1	25262022	C	3.21E-10	2
rs79598985	1	25273023	A	3.36E-06	2
rs188468174	1	25291697	T	3.57E-20	2
rs186127900	1	25318225	T	1.69E-12	2
rs4649041	1	25321754	G	5.95E-14	2
rs115176171	1	25333134	G	4.65E-08	2
rs61776403	1	25361242	A	9.01E-13	2
rs10903128	1	25363338	A	3.97E-08	2
rs79472072	1	25397198	A	7.60E-09	2
rs11586871	1	25399669	T	1.77E-08	2
rs12077761	1	25409256	G	3.45E-07	2
rs12745121	1	25467880	A	5.89E-24	2
rs6600257	1	25472465	A	6.72E-19	2
rs1576101	1	41367198	T	2.29E-08	3
rs6683032	1	47947741	C	2.68E-13	4
rs10888690	1	50960521	C	2.06E-13	5
rs6588396	1	51464565	C	1.01E-10	5
rs67803788	1	118554275	A	4.45E-12	6
rs12083887	1	118881689	A	2.29E-13	6
rs17186024	1	119672649	A	2.08E-16	6
rs12144907	1	152118217	C	4.34E-10	7
rs71635652	1	170224362	G	6.40E-10	8
rs10919382	1	170361164	G	6.39E-24	8
rs2143109	1	170773031	G	1.97E-13	8
rs116315504	1	203790079	T	4.99E-15	9
rs12123537	1	203791174	T	5.87E-15	9
rs78448052	1	203965400	T	4.85E-13	9
rs115282290	1	203979114	T	2.68E-10	9
rs6752754	2	6569566	G	2.83E-14	10
rs844193	2	30626423	T	4.30E-09	11
rs77781418	2	31508999	A	5.60E-15	11
rs77775907	2	31609942	A	3.14E-22	11
rs113923480	2	31610291	T	1.25E-21	11
rs191212334	2	31853354	G	2.63E-01	11

rs13021718	2	32181424	A	1.35E-28	11
rs72787520	2	32851882	G	3.76E-21	11
rs72787535	2	32873774	T	9.20E-21	11
rs72787555	2	32922317	G	2.58E-20	11
rs6732426	2	43587504	C	2.95E-08	12
rs149801367	2	60647456	T	6.85E-07	13
rs745977	2	68081309	A	2.68E-09	14
rs6741945	2	68151619	T	2.08E-06	14
rs6756875	2	68160476	A	1.89E-14	14
rs10928235	2	145678114	T	1.32E-08	15
rs74527054	2	174277357	T	7.90E-08	16
rs13405699	2	174605633	A	1.51E-15	16
rs16862069	2	174607506	C	2.02E-15	16
rs71421546	2	176991857	A	3.13E-19	17
rs6748131	2	177074773	T	2.92E-06	17
rs71421553	2	177086773	A	6.55E-10	17
rs34769088	2	177226167	A	2.09E-07	17
rs6737860	2	177594983	C	1.48E-13	17
rs17668846	2	177616844	C	1.32E-09	17
rs12472159	2	177691543	G	2.65E-10	17
rs10930758	2	177761828	A	1.24E-17	17
rs6755476	2	177858518	C	3.90E-07	17
rs7349332	2	219756383	T	1.52E-21	18
rs192913879	2	219769345	A	2.16E-05	18
rs77177529	2	223070676	T	1.17E-08	19
rs11684254	2	239695893	G	3.68E-45	20
rs57613913	2	239703911	C	4.91E-14	20
rs59712316	2	239729257	G	8.76E-18	20
rs146095395	2	239769885	T	6.87E-11	20
rs12185725	2	239949823	G	1.92E-10	20
rs7621843	3	11603229	G	1.01E-07	21
rs9846246	3	107424090	G	1.10E-10	22
rs115182912	3	125993274	A	6.43E-20	23
rs35892873	3	126068948	T	6.40E-15	23
rs9878451	3	126091660	A	3.21E-11	23
rs6772287	3	138721931	G	1.63E-21	24
rs77438261	3	138997951	G	8.82E-09	24
rs6802174	3	139006664	G	1.45E-11	24
rs7642536	3	139032333	C	5.93E-29	24
rs6440008	3	141154542	C	3.15E-08	25
rs13060333	3	151657769	A	1.37E-18	26
rs1842202	3	151754768	G	1.54E-11	26
rs13092705	3	182237032	G	2.06E-07	27
rs7680591	4	81197949	A	3.15E-27	28
rs6811219	4	81251198	C	7.65E-11	28
rs12509636	4	106010433	T	1.03E-08	29
rs10516537	4	107628071	A	1.60E-08	30
rs4109346	4	107888788	A	1.77E-08	30

rs76067940	4	108052995	T	4.10E-10	30
rs1351637	5	44441703	G	2.93E-09	31
rs35558461	5	67193451	A	8.08E-06	32
rs4976028	5	67290733	G	1.66E-09	32
rs186749	5	122454305	A	2.00E-08	33
rs77239429	5	158305074	T	8.92E-11	34
rs17543668	5	158311643	A	1.04E-09	34
rs1422798	5	158320877	G	8.19E-44	34
rs17626412	5	158338972	A	4.20E-14	34
rs17718288	5	158488326	G	1.36E-07	34
rs62390189	6	327331	A	4.16E-01	35
rs2671427	6	385735	T	1.46E-08	35
rs12203592	6	396321	T	2.21E-35	35
rs9392026	6	453415	G	6.26E-09	35
rs9392563	6	470930	G	5.87E-04	35
rs11242982	6	537157	T	5.62E-10	35
rs11243290	6	9000423	A	2.78E-08	36
rs78576899	6	9304649	T	2.05E-09	36
rs9357047	6	9327556	C	3.57E-35	36
rs2294736	6	9389312	C	2.42E-07	36
rs6457977	6	9420099	A	4.85E-15	36
rs79032435	6	9464547	T	8.16E-18	36
rs114190032	6	9509256	T	7.09E-11	36
rs139092879	6	9538124	A	1.29E-07	36
rs855256	6	9605188	T	6.85E-23	36
rs6939066	6	9725270	T	2.09E-10	36
rs141173114	6	10186332	T	1.40E-08	36
rs11759950	6	10226233	T	4.30E-07	36
rs9398035	6	106041382	C	9.48E-12	37
rs4946689	6	106201608	G	2.49E-10	37
rs12214131	6	106207921	A	6.73E-17	37
rs2344704	6	111965138	A	7.07E-09	38
rs4363059	6	126466605	A	1.00E-11	39
rs9388490	6	126704795	T	2.87E-22	39
rs199961668	7	510890	AC	2.13E-05	40
rs9691699	7	533352	C	1.28E-21	40
rs150909339	7	552468	C	2.46E-06	40
rs9801230	7	564029	T	3.90E-11	40
rs9801466	7	564096	A	2.15E-13	40
rs9330381	7	565948	G	1.24E-08	40
rs801525	7	18710388	A	6.77E-10	41
rs56349397	7	18865362	C	4.42E-10	41
rs117584427	7	18887633	G	1.51E-07	41
rs71530654	7	18896988	G	6.79E-69	41
rs77527369	7	18925499	A	1.62E-09	41
rs10278449	7	18931219	C	2.87E-13	41
rs73071393	7	19096224	G	5.30E-06	41
rs10225279	7	19133072	T	4.65E-13	41

rs58788673	7	46904299	TCA	1.03E-16	42
rs939963	7	68587797	G	3.49E-53	43
rs12540951	7	68623892	A	1.27E-15	43
rs1195215	7	68736093	C	4.62E-12	43
rs4718886	7	68898689	G	8.89E-31	43
rs6943067	7	68908285	A	4.56E-12	43
rs117463770	7	69607509	G	1.55E-06	43
rs1474368	7	69707623	G	2.06E-09	43
rs9719620	7	130994380	T	2.38E-10	44
rs10808813	8	77516942	G	7.19E-05	45
rs76729432	8	108805685	C	1.81E-11	46
rs145562694	8	108818496	A	2.03E-17	46
rs2047198	8	108962627	C	8.24E-18	46
rs34657911	8	109093743	A	5.68E-11	46
rs117769774	8	109102918	A	1.97E-12	46
rs79206101	8	109145555	T	7.16E-29	46
rs77767830	8	109268396	T	3.02E-13	46
rs118013985	8	109394188	C	1.62E-10	46
rs77096234	8	109657430	A	1.03E-20	46
rs183207557	8	109682947	T	2.97E-01	46
rs55908337	8	109872393	T	2.27E-14	46
rs75520281	8	109974922	G	2.26E-15	46
rs800890	8	116439639	T	2.82E-06	47
rs6982226	8	117015142	G	4.53E-10	47
rs58833541	8	117111910	G	8.14E-07	47
rs998245	8	117239874	T	6.43E-07	47
rs73667301	9	109628051	G	3.03E-08	48
rs1906457	10	62938418	T	1.73E-08	49
rs1873465	10	78189959	C	3.50E-10	50
rs1907350	10	78194812	G	6.12E-24	50
rs11497798	10	78377170	C	8.21E-15	50
rs11594897	10	78377573	G	1.42E-18	50
rs3781452	10	126355129	C	1.66E-21	51
rs78321654	11	27453319	A	1.53E-08	52
rs2863081	11	44431807	G	6.35E-18	53
rs174581	11	61606683	A	7.08E-08	54
rs11220486	11	126291021	A	1.37E-06	55
rs7974900	12	26428677	T	7.04E-20	56
rs73072491	12	26438450	G	3.95E-11	56
rs10843003	12	27986940	G	1.06E-08	57
rs11049231	12	28091700	A	5.43E-08	57
rs10771462	12	29157328	G	1.88E-16	58
rs74895226	12	29239638	T	4.03E-06	58
rs76972608	12	130563363	T	2.19E-16	59
rs72662240	14	30562926	A	4.30E-08	60
rs8013382	14	95208412	G	3.09E-09	61
rs997683	15	57541904	A	1.13E-09	62
rs2028122	15	60841275	A	1.77E-08	63

rs2117234	15	70039200	T	6.52E-18	64
rs12902958	15	70040254	A	1.38E-17	64
rs2074585	15	91009484	G	1.33E-13	65
rs246180	16	14391923	A	1.77E-16	66
rs12447206	16	85319399	C	7.61E-07	67
rs72809169	17	12457050	G	6.57E-11	68
rs143942024	17	43926458	T	1.42E-02	69
rs140843301	17	43939214	C	2.39E-03	69
rs112385572	17	44066172	G	3.96E-32	69
rs538628	17	44787313	C	7.10E-33	69
rs62060349	17	55231168	C	2.13E-23	70
rs145226407	17	55231601	G	4.15E-12	70
rs29073	18	9971790	C	7.10E-11	71
rs427754	18	10361777	T	8.99E-07	71
rs206447	18	10439986	G	3.78E-11	71
rs1276250	18	42440538	T	3.70E-10	72
rs12326164	18	42676928	A	2.93E-09	72
rs8085664	18	42814156	A	1.69E-28	72
rs11659559	18	53005934	A	1.49E-06	73
rs17594358	18	53050489	G	3.04E-07	73
rs8095770	18	53093724	C	6.21E-07	73
rs78803556	18	53118462	A	2.41E-06	73
rs7226979	18	60924970	T	3.16E-08	74
rs6047620	20	21761183	T	3.42E-16	75
rs6113382	20	21787568	A	2.19E-10	75
rs6035986	20	21894764	G	4.64E-91	75
rs62219289	20	21988334	G	2.47E-08	75
rs78297031	20	21989602	C	4.22E-07	75
rs201593	20	22033819	G	3.36E-110	75
rs12625546	20	22037409	T	5.07E-11	75
rs75434917	20	22043888	T	1.57E-19	75
rs17752365	20	22047324	A	4.69E-09	75
rs7362397	20	22100070	T	1.33E-68	75
rs7362398	20	22100072	T	1.33E-68	75
rs79284724	20	22106657	G	1.74E-10	75
rs199791	20	22284321	C	3.01E-26	75
rs199823	20	22323858	C	2.17E-13	75
rs17265513	20	39832628	C	3.87E-14	76
rs6025176	20	55390633	C	2.95E-09	77
rs985546	20	55434683	C	3.34E-13	77
rs68088846	21	36208167	A	9.13E-17	78
rs11701104	21	36238517	T	1.96E-08	78
rs75732647	21	44626739	T	9.81E-10	79
rs112348497	21	44627879	GC	1.52E-10	79
rs141433484	21	45936195	A	1.29E-06	80
rs690333	21	46172675	C	2.42E-08	80
rs5934505	X	8913826	C	1.07E-15	81
rs140707533	X	54624388	A	3.47E-15	82

rs138216499	X	55330959	A	1.41E-12	82
rs73206583	X	56287726	A	7.51E-07	82
rs139470886	X	57309130	A	1.12E-18	83
rs147829649	X	58005480	G	2.31E-31	83
rs73209413	X	58097007	C	4.70E-10	83
rs5989004	X	58194651	T	9.44E-13	83
rs140914450	X	62062376	G	5.15E-19	84
rs111810219	X	62937827	A	1.09E-11	84
rs146112277	X	63755444	A	3.05E-12	84
rs6624142	X	64405169	C	4.63E-13	84
rs143755874	X	64859400	A	9.41E-09	84
rs5965019	X	64993425	A	2.65E-12	84
rs138876904	X	65052140	T	2.11E-09	84
rs147154263	X	65083247	T	3.43E-61	84
rs73213393	X	65217956	A	9.06E-22	84
rs147670940	X	65252953	T	8.88E-13	84
rs145867342	X	65541956	T	2.96E-49	84
rs112069404	X	65551148	A	1.11E-22	84
rs79798752	X	65632419	C	4.30E-15	84
rs73221553	X	65926042	A	2.44E-10	84
rs73221556	X	65933285	A	0.00E+00	84
rs144393202	X	66114131	A	2.03E-14	84
rs17216820	X	66337545	T	1.20E-29	84
rs146636673	X	66460331	C	1.14E-08	84
rs12558842	X	66481800	C	0.00E+00	84
rs142169094	X	66555112	G	5.20E-14	84
rs73227823	X	66566037	G	2.42E-12	84
rs62604342	X	66701867	G	7.29E-09	84
rs113222435	X	66963707	T	1.59E-12	84
rs5919427	X	67003584	C	1.19E-191	84
rs148652266	X	67139063	A	1.92E-50	84
rs73212804	X	67158738	T	7.05E-11	84
rs17302236	X	67174031	C	1.37E-26	84
rs7061504	X	67363801	G	3.54E-36	84
rs73212868	X	67425333	A	5.02E-26	84
rs140488081	X	67496002	T	6.28E-63	84
rs5965561	X	67595914	C	4.10E-19	84
rs11796997	X	152759861	A	2.22E-08	85

SNPs were aligned based on the GRCh37 genome assembly. BP, base pair; EA, effect allele.

* SNPs were previously identified with genome-wide MPB association in 52,874 male participants of the UKBB by Hagenaars et al. PLoS Genet. 2017;13(2):e1006594, which largely overlaps with the 55,573 UKBB males used here for feature selection.

Supplementary Table 2. Accumulative AUC in the model testing dataset of 26,177 UKBB males based on the 117 most MPB predictive SNPs obtained from feature selection analysis of 270 SNPs in the UKBB feature selection dataset.

				AUC				
Rank	Predictor	CHR	BP	No hair loss	Slight hair loss	Moderate hair loss	Severe hair loss	Any *vs.* no hair loss
---	Age	---	---	0.560	0.566	0.579	0.562	0.560
1	rs12558842	X	66481800	0.608	0.568	0.593	0.604	0.608
2	rs201593	20	22033819	0.620	0.570	0.601	0.611	0.620
3	rs71530654	7	18896988	0.625	0.571	0.602	0.619	0.625
4	rs7542354	1	11040385	0.631	0.571	0.602	0.625	0.631
5	rs939963	7	68587797	0.636	0.571	0.603	0.629	0.636
6	rs7642536	3	139032333	0.638	0.571	0.604	0.635	0.638
7	rs11684254	2	239695893	0.641	0.572	0.606	0.639	0.641
8	rs1422798	5	158320877	0.645	0.572	0.607	0.644	0.645
9	rs77177529	2	223070676	0.646	0.578	0.608	0.644	0.646
10	rs7061504	X	67363801	0.649	0.578	0.608	0.647	0.649
11	rs9357047	6	9327556	0.650	0.579	0.608	0.651	0.650
12	rs538628	17	44787313	0.654	0.579	0.609	0.655	0.654
13	rs12203592	6	396321	0.656	0.580	0.611	0.658	0.656
14	rs13021718	2	32181424	0.657	0.580	0.611	0.659	0.657
15	rs79206101	8	109145555	0.659	0.580	0.612	0.660	0.659
16	rs7680591	4	81197949	0.660	0.580	0.612	0.661	0.660
17	rs4718886	7	68898689	0.662	0.580	0.613	0.663	0.661
18	rs10919382	1	170361164	0.661	0.581	0.613	0.665	0.661
19	rs6802174	3	139006664	0.661	0.582	0.616	0.667	0.661
20	rs8085664	18	42814156	0.662	0.582	0.616	0.670	0.662
21	rs1907350	10	78194812	0.664	0.583	0.616	0.671	0.665
22	rs6772287	3	138721931	0.665	0.584	0.616	0.673	0.665
23	rs12745121	1	25467880	0.667	0.584	0.617	0.675	0.667
24	rs62060349	17	55231168	0.668	0.584	0.617	0.676	0.668
25	rs3781452	10	126355129	0.670	0.585	0.617	0.679	0.670
26	rs7974900	12	26428677	0.671	0.585	0.619	0.680	0.671
27	rs188468174	1	25291697	0.673	0.585	0.619	0.681	0.673
28	rs9388490	6	126704795	0.673	0.586	0.620	0.682	0.673
29	rs7349332	2	219756383	0.674	0.586	0.620	0.682	0.674
30	rs9691699	7	533352	0.675	0.586	0.621	0.682	0.675
31	rs115182912	3	125993274	0.676	0.587	0.621	0.684	0.676
32	rs13060333	3	151657769	0.677	0.586	0.621	0.685	0.677
33	rs71421546	2	176991857	0.678	0.586	0.621	0.686	0.678
34	rs855256	6	9605188	0.679	0.587	0.622	0.687	0.679
35	rs58788673	7	46904299	0.680	0.587	0.622	0.687	0.680
36	rs114190032	6	9509256	0.680	0.587	0.623	0.689	0.680
37	rs2117234	15	70039200	0.681	0.587	0.623	0.689	0.681
38	rs17186024	1	119672649	0.681	0.588	0.623	0.691	0.681
39	rs5934505	X	8913826	0.682	0.587	0.622	0.692	0.682
40	rs10930758	2	177761828	0.683	0.588	0.623	0.693	0.683

41	rs76972608	12	130563363	0.683	0.589	0.623	0.694	0.683
42	rs35892873	3	126068948	0.684	0.589	0.623	0.694	0.684
43	rs2863081	11	44431807	0.684	0.590	0.624	0.695	0.685
44	rs68088846	21	36208167	0.685	0.589	0.624	0.696	0.685
45	rs6752754	2	6569566	0.686	0.589	0.624	0.698	0.686
46	rs12214131	6	106207921	0.687	0.589	0.625	0.698	0.687
47	rs985546	20	55434683	0.687	0.589	0.625	0.699	0.687
48	rs34657911	8	109093743	0.688	0.590	0.625	0.701	0.688
49	rs10928235	2	145678114	0.690	0.592	0.626	0.702	0.690
50	rs10225279	7	19133072	0.690	0.592	0.626	0.702	0.690
51	rs10771462	12	29157328	0.691	0.593	0.626	0.703	0.691
52	rs745977	2	68081309	0.692	0.593	0.627	0.703	0.692
53	rs246180	16	14391923	0.693	0.594	0.628	0.704	0.693
54	rs10888690	1	50960521	0.693	0.594	0.628	0.705	0.693
55	rs2074585	15	91009484	0.694	0.594	0.628	0.705	0.694
56	rs17302236	X	67174031	0.695	0.594	0.629	0.707	0.695
57	rs9719620	7	130994380	0.696	0.594	0.628	0.708	0.696
58	rs17265513	20	39832628	0.696	0.595	0.629	0.709	0.696
59	rs13405699	2	174605633	0.697	0.596	0.629	0.710	0.697
60	rs77767830	8	109268396	0.697	0.596	0.629	0.711	0.697
61	rs6982226	8	117015142	0.698	0.596	0.630	0.711	0.698
62	rs12123537	1	203791174	0.698	0.596	0.629	0.712	0.698
63	rs6683032	1	47947741	0.698	0.596	0.629	0.712	0.698
64	rs112348497	21	44627879	0.699	0.596	0.629	0.713	0.699
65	rs29073	18	9971790	0.699	0.597	0.629	0.713	0.699
66	rs1351637	5	44441703	0.699	0.597	0.629	0.713	0.699
67	rs2143109	1	170773031	0.700	0.597	0.630	0.713	0.700
68	rs9846246	3	107424090	0.700	0.597	0.630	0.714	0.700
69	rs186749	5	122454305	0.701	0.597	0.630	0.714	0.701
70	rs11243290	6	9000423	0.702	0.598	0.630	0.715	0.702
71	rs12083887	1	118881689	0.702	0.598	0.631	0.716	0.702
72	rs998245	8	117239874	0.702	0.597	0.631	0.716	0.702
73	rs72809169	17	12457050	0.703	0.598	0.631	0.716	0.703
74	rs76067940	4	108052995	0.702	0.599	0.631	0.717	0.703
75	rs997683	15	57541904	0.703	0.599	0.631	0.717	0.703
76	rs427754	18	10361777	0.703	0.599	0.631	0.718	0.703
77	rs844193	2	30626423	0.703	0.599	0.631	0.718	0.704
78	rs6440008	3	141154542	0.704	0.599	0.631	0.718	0.704
79	rs12902958	15	70040254	0.704	0.599	0.631	0.719	0.704
80	rs1906457	10	62938418	0.705	0.599	0.631	0.719	0.705
81	rs142020459	1	10572083	0.704	0.599	0.631	0.720	0.704
82	rs78321654	11	27453319	0.705	0.599	0.631	0.720	0.705
83	rs8013382	14	95208412	0.705	0.599	0.631	0.721	0.705
84	rs12144907	1	152118217	0.705	0.600	0.631	0.721	0.705
85	rs72662240	14	30562926	0.705	0.600	0.632	0.721	0.705
86	rs113923480	2	31610291	0.706	0.600	0.632	0.721	0.705
87	rs17594358	18	53050489	0.705	0.600	0.632	0.722	0.705
88	rs12185725	2	239949823	0.706	0.600	0.632	0.722	0.706

89	rs4946689	6	106201608	0.706	0.601	0.632	0.722	0.706
90	rs6737860	2	177594983	0.707	0.601	0.632	0.722	0.706
91	rs1842202	3	151754768	0.707	0.600	0.632	0.722	0.706
92	rs4976028	5	67290733	0.707	0.600	0.632	0.723	0.706
93	rs6756875	2	68160476	0.707	0.600	0.632	0.723	0.707
94	rs73667301	9	109628051	0.707	0.600	0.632	0.723	0.707
95	rs73212868	X	67425333	0.707	0.601	0.632	0.723	0.707
96	rs11049231	12	28091700	0.708	0.601	0.633	0.724	0.708
97	rs62604342	X	66701867	0.708	0.600	0.633	0.724	0.708
98	rs147670940	X	65252953	0.708	0.600	0.633	0.724	0.708
99	rs79032435	6	9464547	0.708	0.601	0.633	0.724	0.708
100	rs7621843	3	11603229	0.709	0.601	0.633	0.724	0.708
101	rs11586871	1	25399669	0.709	0.601	0.633	0.725	0.709
102	rs147829649	X	58005480	0.709	0.601	0.633	0.725	0.709
103	rs140707533	X	54624388	0.709	0.601	0.633	0.725	0.709
104	rs2344704	6	111965138	0.709	0.601	0.633	0.725	0.709
105	rs12509636	4	106010433	0.709	0.601	0.633	0.725	0.709
106	rs2028122	15	60841275	0.709	0.601	0.633	0.725	0.709
107	rs11796997	X	152759861	0.709	0.602	0.634	0.725	0.709
108	rs1576101	1	41367198	0.709	0.601	0.634	0.726	0.709
109	rs690333	21	46172675	0.709	0.601	0.634	0.726	0.709
110	rs6732426	2	43587504	0.710	0.602	0.634	0.727	0.710
111	rs7226979	18	60924970	0.710	0.602	0.634	0.727	0.710
112	rs174581	11	61606683	0.711	0.602	0.634	0.727	0.710
113	rs13092705	3	182237032	0.711	0.602	0.635	0.728	0.711
114	rs149801367	2	60647456	0.711	0.602	0.635	0.728	0.711
115	rs12447206	16	85319399	0.711	0.602	0.635	0.728	0.711
116	rs11220486	11	126291021	0.711	0.602	0.635	0.728	0.711
117	rs10808813	8	77516942	0.711	0.602	0.635	0.728	0.711

Supplementary Table 3. 107 of the 117 SNPs used for external MPB prediction model validation in the Bonn Study and their imputation quality in the Bonn Study.

SNP	CHR	BP	Imputation-info
rs142020459	1	10572083	0.563
rs7542354	1	11040385	0.902
rs11586871	1	25399669	0.843
rs12745121	1	25467880	0.905
rs1576101	1	41367198	0.972
rs6683032	1	47947741	0.897
rs10888690	1	50960521	0.932
rs12083887	1	118881689	0.973
rs17186024	1	119672649	0.995
rs12144907	1	152118217	0.926
rs10919382	1	170361164	0.936
rs2143109	1	170773031	1
rs6752754	2	6569566	0.667
rs844193	2	30626423	0.967
rs113923480	2	31610291	0.781
rs13021718	2	32181424	0.977
rs6732426	2	43587504	0.975
rs149801367	2	60647456	0.959
rs745977	2	68081309	0.959
rs6756875	2	68160476	0.976
rs10928235	2	145678114	0.915
rs13405699	2	174605633	0.479
rs71421546	2	176991857	0.807
rs6737860	2	177594983	0.656
rs10930758	2	177761828	0.975
rs7349332	2	219756383	0.866
rs77177529	2	223070676	0.849
rs11684254	2	239695893	0.628
rs12185725	2	239949823	0.886
rs7621843	3	11603229	0.789
rs9846246	3	107424090	1
rs115182912	3	125993274	0.728
rs35892873	3	126068948	0.976
rs6772287	3	138721931	0.995
rs6802174	3	139006664	0.978
rs7642536	3	139032333	0.869
rs6440008	3	141154542	0.86
rs13060333	3	151657769	0.991
rs1842202	3	151754768	0.997
rs7680591	4	81197949	0.697
rs12509636	4	106010433	0.934
rs76067940	4	108052995	0.533
rs1351637	5	44441703	0.841
rs4976028	5	67290733	0.665

rs186749	5	122454305	0.984
rs1422798	5	158320877	0.984
rs11243290	6	9000423	0.917
rs9357047	6	9327556	0.99
rs79032435	6	9464547	0.877
rs114190032	6	9509256	0.822
rs855256	6	9605188	0.99
rs4946689	6	106201608	0.884
rs12214131	6	106207921	0.946
rs2344704	6	111965138	0.867
rs9388490	6	126704795	0.98
rs71530654	7	18896988	0.862
rs10225279	7	19133072	0.4
rs58788673	7	46904299	0.977
rs939963	7	68587797	0.97
rs4718886	7	68898689	0.757
rs9719620	7	130994380	0.974
rs10808813	8	77516942	0.83
rs34657911	8	109093743	0.98
rs79206101	8	109145555	0.833
rs77767830	8	109268396	0.677
rs6982226	8	117015142	0.901
rs998245	8	117239874	0.406
rs73667301	9	109628051	0.935
rs1906457	10	62938418	0.89
rs1907350	10	78194812	1
rs3781452	10	126355129	0.964
rs78321654	11	27453319	0.871
rs2863081	11	44431807	0.982
rs174581	11	61606683	0.963
rs11220486	11	126291021	0.684
rs7974900	12	26428677	0.965
rs11049231	12	28091700	0.929
rs10771462	12	29157328	0.963
rs76972608	12	130563363	0.914
rs72662240	14	30562926	0.838
rs8013382	14	95208412	0.974
rs997683	15	57541904	0.949
rs2028122	15	60841275	1
rs2117234	15	70039200	0.986
rs12902958	15	70040254	0.964
rs2074585	15	91009484	0.813
rs246180	16	14391923	0.508
rs72809169	17	12457050	0.779
rs538628	17	44787313	0.977
rs62060349	17	55231168	0.982
rs29073	18	9971790	0.785
rs8085664	18	42814156	0.854

rs17594358	18	53050489	0.883
rs7226979	18	60924970	0.771
rs201593	20	22033819	0.932
rs17265513	20	39832628	0.906
rs68088846	21	36208167	0.878
rs112348497	21	44627879	0.602
rs690333	21	46172675	0.729
rs5934505	X	8913826	0.998
rs140707533	X	54624388	0.604
rs147670940	X	65252953	0.853
rs12558842	X	66481800	0.885
rs62604342	X	66701867	0.475
rs17302236	X	67174031	0.482
rs7061504	X	67363801	0.942
rs11796997	X	152759861	0.861

Supplementary Table 4. Prediction accuracies of a 10-SNP* MPB prediction model for no versus slight/moderate/severe hair loss as obtained from the UKBB model testing dataset; the model was built in the UKBB model training dataset.

	Without Age	With Age
**AUC**	0.572	0.611
**SENS**	0.949	0.646
**SPEC**	0.070	0.492
**PPV**	0.324	0.374
**NPV**	0.744	0.747

* 10 available of the 11 SNPs previously used for MPB prediction modelling by Liu et al. Eur J Hum Genet. 2016;24(6):895-902.

Supplementary Table 5. Prediction confusion matrix of a 10-SNP* MPB prediction model for no versus slight/moderate/severe hair loss as obtained from the UKBB model testing dataset; the model was built in the UKBB model training dataset.

Truth\Predicted	Without Age		With Age	
	No hair loss	Slight/Moderate/Severe hair loss	No hair loss	Slight/Moderate/Severe hair loss
**No hair loss**	7944	428	5411	2961
**Slight/Moderate/Severe hair loss**	16561	1244	9045	8760

* 10 available of the 11 SNPs previously used for MPB prediction modelling by Liu et al. Eur J Hum Genet. 2016;24(6):895-902.

Supplementary Figure 1. Overview on study design.

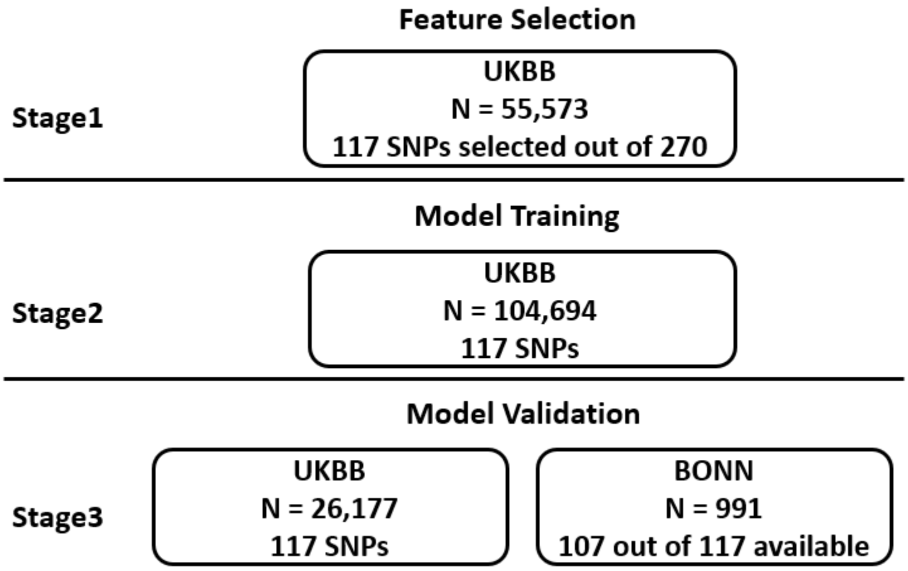

The 55,573 UKBB men used as marker ascertainment dataset in Stage 1 for feature selection include the 52,874 UKBB men Hagenaars et al. used for discovering the MPB-association of the 270 SNPs applied for feature selection. The model training dataset of UKBB in Stage 2 was independent of the UKBB data sets used for marker ascertainment in Stage 1 and model testing in Stage 3. The model validation step in Stage 3 was split into an internal validation of the 117-SNP model in the UKBB model testing dataset based on UKBB samples not used for marker ascertainment in Stage 1 and model training in Stage 2, and an external validation of the partial 107-SNP model in the Bonn Study; this 107-SNP model was trained and internally validated in the same UKBB samples used in Stage 2 and 3.

Supplementary Figure 2. Male baldness patterns adapted from the Hamilton-Norwood scale. (A) Male baldness pattern classification as used in the UKBB study; (B) Conversion from Hamilton-Norwood scale to 4-categories male baldness pattern classification used in the Bonn Study.

(A)

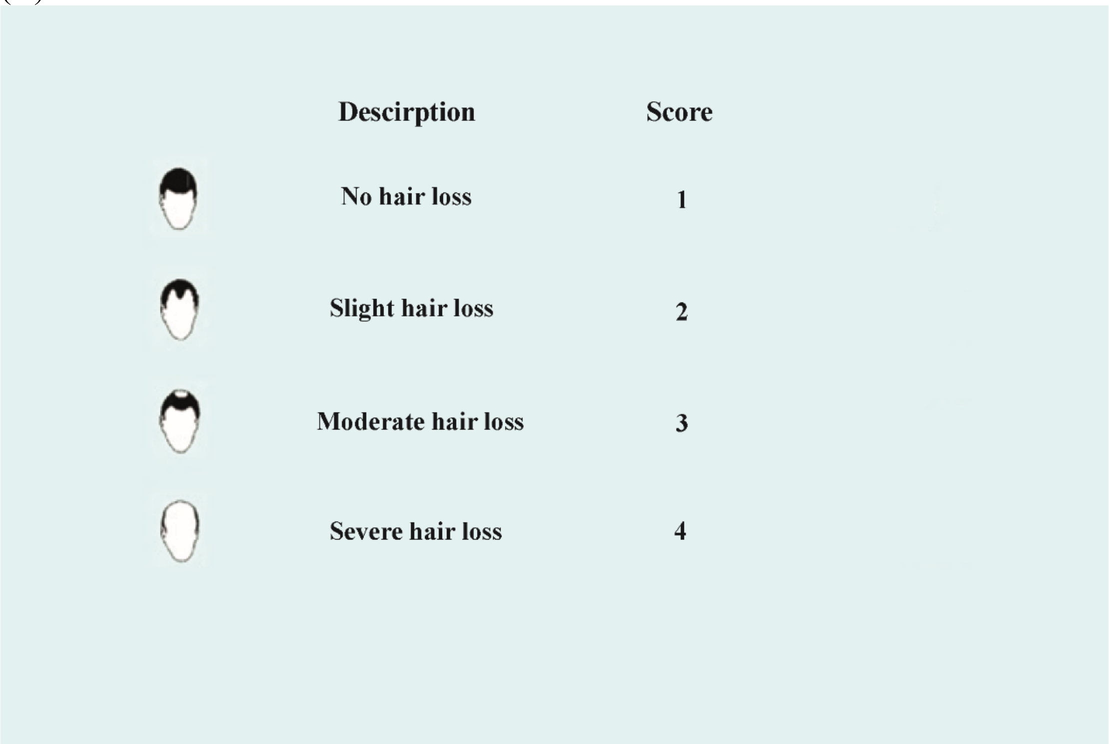

(B)

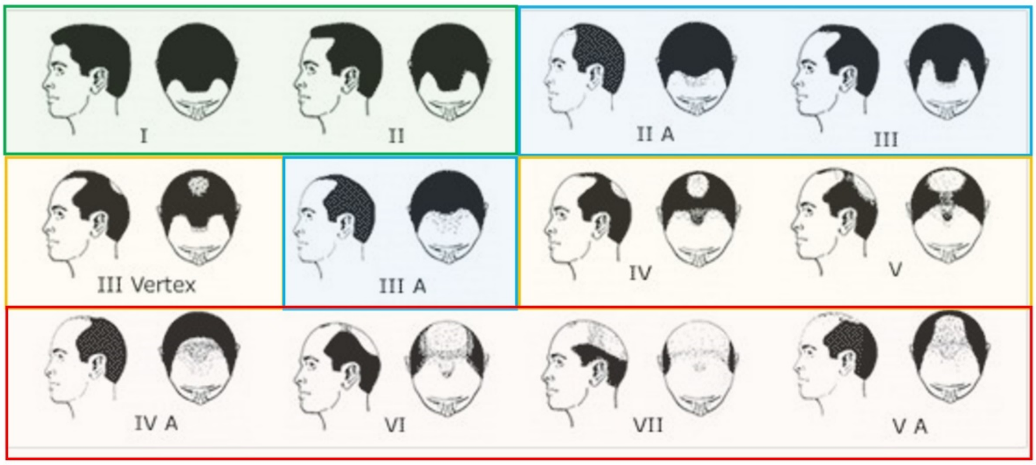

1: I, II - (no or mild frontal)
2: IIa, III, IIIa (moderate frontal)
3: 3vertex, IV, V (vertex)
4: IVa, Va, VI, VII (frontal and vertex)

Supplementary Figure 3. Age distribution and the percentage of each baldness category in each age group among all 186,444 UKBB males and 911 Bonn Study males, respectively.

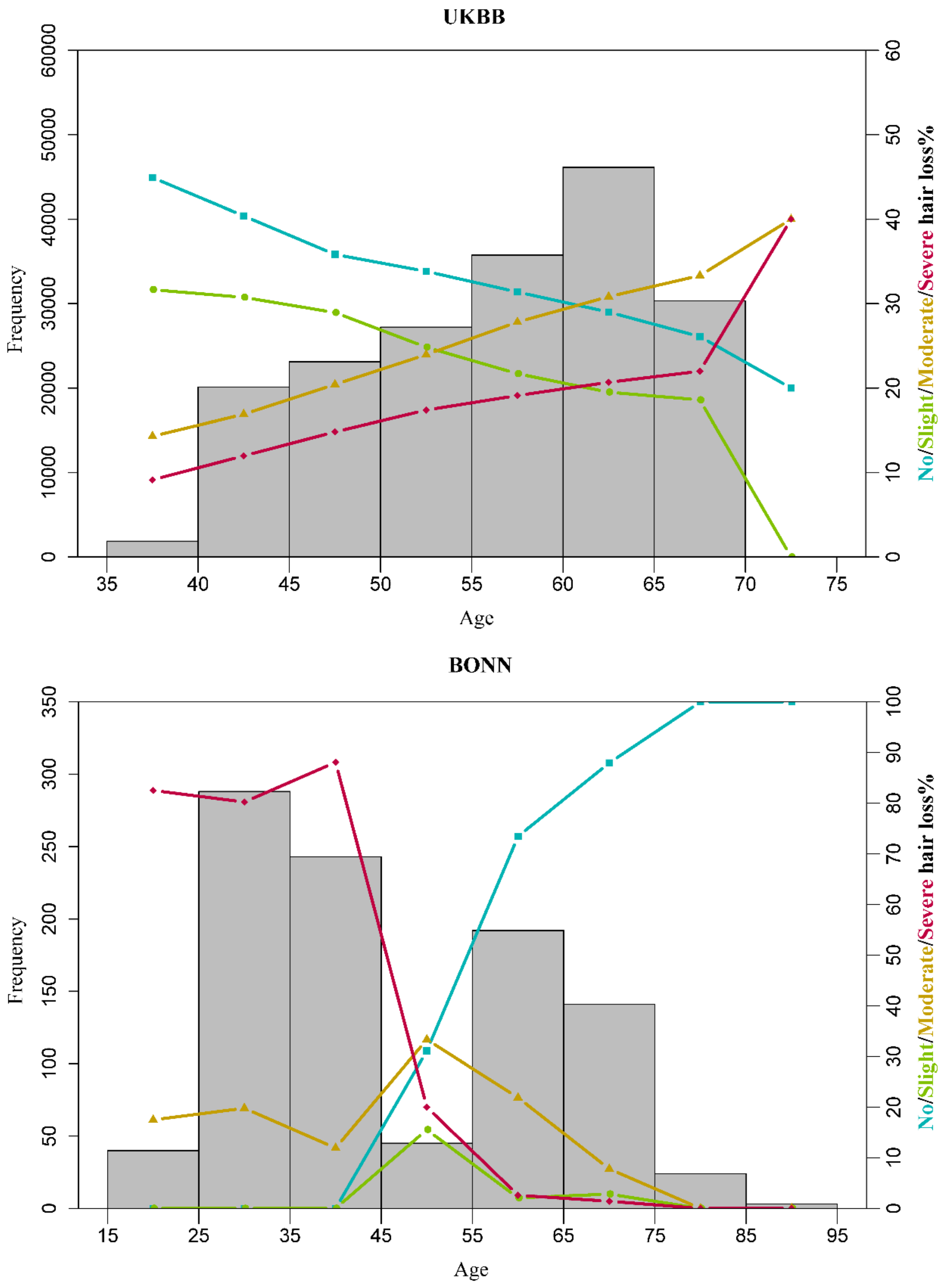

Supplementary Figure 4. Prediction accuracy expressed as AUC of the 4-category genetic MLR prediction models for MPB based on 117 SNP predictors with and without considering age as additional predictor for 6 age groups from the model validation dataset of 26,177 UKBB males.

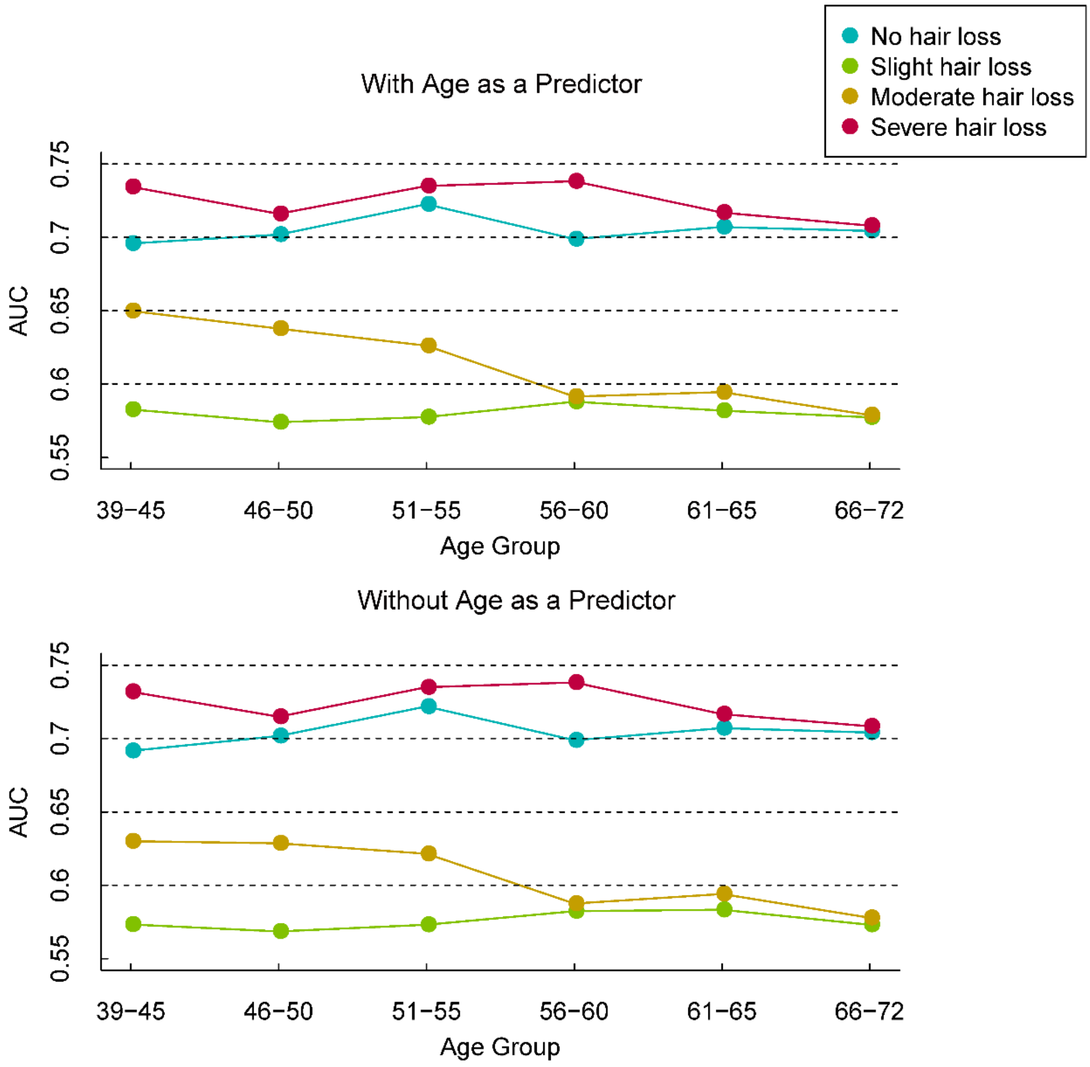

Supplement: Supplementary file 1 — Supplementary Information [file 41431_2022_1201_MOESM1_ESM.pdf]
